# Breastfeeding in infancy confers sex-specific, long-term protection against metabolic dysfunction-associated steatohepatitis and adverse liver outcomes

**DOI:** 10.1186/s13293-026-00919-4

**Published:** 2026-05-12

**Authors:** Huiyi Chen, Haobin Chen, Sheng Shen, Xinfeng Ji, Huiling Deng, Qian Wang, Qingying Zhang, Maolong Dong, Jun Ren, Guifang Hu, Jian Sun, Kaifeng Wang

**Affiliations:** 1 Department of Infectious Diseases, The First Affiliated Hospital of Shantou University Medical College, Shantou, 515041 China; 2 Department of Burns, Nanfang Hospital, Southern Medical University, Guangzhou, 510515 China; 3 Department of Preventive Medicine, Shantou University Medical College, No. 21, Xinling Road, Shantou, Shantou, 515041 China; 4 State Key Laboratory of Multi-organ Injury Prevention and Treatment, Key Laboratory of Infectious Diseases Research in South China, Guangdong Provincial Key Laboratory of Viral Hepatitis Research, Guangdong Provincial Clinical Research Center for Viral Hepatitis, Guangdong Institute of Hepatology, Department of Infectious Diseases, Nanfang Hospital, Ministry of Education, Southern Medical University, Guangzhou, 510515 China; 5 Department of Research, The First Affiliated Hospital of Shantou University Medical College, Shantou, 515041 China; 6 Department of Epidemiology, School of Public Health, Southern Medical University, Guangzhou, 510515 China; 7 Department of Burns, Tongren Hospital of Wuhan University & Wuhan Third Hospital, Wuhan, 430060 China; 8 Department of Cardiology, Shanghai Institute of Cardiovascular Diseases, Zhongshan Hospital Fudan University, Shanghai, 200032 China; 9 National Clinical Research Center for Interventional Medicine, Shanghai, 200032 China

**Keywords:** Breast Feeding, Non‑alcoholic Fatty Liver Disease, Sex Characteristics, Liver Cirrhosis, Liver Neoplasms, Insulin-Like Growth Factor I

## Abstract

**Background:**

It remains unclear whether breastfeeding in infancy is associated with risk of metabolic dysfunction-associated steatohepatitis (MASH) and adverse liver outcomes in adulthood.

**Methods:**

We analyzed data from the prospective UK Biobank. Breastfeeding in infancy was ascertained via self-report. In a cross‑sectional analysis involving 26,850 participants with liver MRI data, metabolic dysfunction-associated steatotic liver disease (MASLD) was defined as PDFF > 5.5%, and MASH as PDFF > 5.5% combined with cT1 > 800 ms. Additionally, a longitudinal analysis of 378,702 participants assessed associations with incident cirrhosis, hepatocellular carcinoma (HCC), and liver‑related mortality.

**Results:**

In MRI-based analysis, breastfeeding in infancy was associated with lower odds of MASLD (OR 0.89, 95% CI 0.80–0.99) and MASH (OR 0.79, 95% CI 0.65–0.97) in females but not males, with a significant sex interaction for MASH (P for interaction = 0.023) but not for MASLD (P for interaction = 0.301). Over a median follow‑up of 14.9 years, breastfeeding was associated with reduced risks of cirrhosis (HR 0.71, 95% CI 0.60–0.83), HCC (HR 0.52, 95% CI 0.33–0.82), and liver‑related mortality (HR 0.63, 95% CI 0.46–0.86) in females. No such associations were observed in males (P for interaction = 0.047, 0.030, and 0.008, respectively). Mediation analysis revealed that circulating insulin-like growth factor-1 (IGF-1) mediated the association specifically in females, with a sex difference in the indirect effect (FDR < 0.001).

**Conclusions:**

Breastfeeding in infancy confers a long-term protective effect against MASH and adverse liver outcomes in women, but not in men. This sex-specific protective association was mediated by circulating IGF-1, suggesting a potential underlying mechanism.

**Supplementary Information:**

The online version contains supplementary material available at 10.1186/s13293-026-00919-4.

## Highlights

Among participants with liver MRI data, breastfeeding in infancy was associated with lower odds of MRI‑defined MASH in women, but not in men.

Prospective analysis of 378,702 individuals (median 14.9-year follow-up) confirmed that breastfeeding was linked to reduced risks of cirrhosis, HCC, and liver-related mortality in women, but not in men.

Mediation analysis indicated that the protective effect in women may be partially explained by higher circulating IGF‑1 levels, suggesting a plausible biological pathway.

The robustness of these associations is supported by consistent results across sensitivity analyses and the absence of an association with accidental mortality.

**Supplementary Information:**

The online version contains supplementary material available at 10.1186/s13293-026-00919-4.

## Introduction

Metabolic dysfunction-associated steatotic liver disease (MASLD), characterized by pathological lipid accumulation in hepatocytes, affects approximately 30% of the global population and has become the most prevalent form of chronic liver disease worldwide [1]. Its progressive inflammatory subtype, metabolic dysfunction-associated steatohepatitis (MASH), involves hepatocellular injury and inflammation and develops in an estimated 20–30% of individuals with MASLD [2]. While simple steatosis typically follows a benign clinical course, patients with MASH face substantially elevated risks of cirrhosis, hepatocellular carcinoma (HCC), and liver-related mortality. MASH has emerged as a leading indication for liver transplantation, particularly in high-income countries [3, 4]. Although resmetirom was recently approved by the US Food and Drug Administration as the first targeted therapy for MASH with fibrosis, only about one-quarter of treated patients achieve clinical benefit [5, 6]. These limitations highlight the need for novel strategies to prevent MASH onset and progression.

Early-life nutrition, particularly breastfeeding during infancy, is a potentially modifiable factor. Beyond its well‑established benefits for infant health, breastfeeding has been linked to lower risks of several adult‑onset conditions, including depression, type 2 diabetes and certain cancers [7–12]. However, whether breastfeeding in infancy is associated with the risk of MASH and adverse liver outcomes in adulthood remains unknown. Furthermore, several studies indicate that the long-term metabolic effects of early‑life nutrition might differ between males and females. For instance, exposure to famine in early life has been associated with a higher prevalence of fatty liver disease in women but not in men [13]. Likewise, maternal dietary interventions during lactation improve insulin sensitivity, specifically in female offspring [13, 14]. Additionally, insulin‑like growth factor 1 (IGF‑1) is a hepatoprotective molecule that responds to early‑life nutrition in a sex‑specific manner [15–17]. Together, these findings raise the hypothesis that the long‑term effects of breastfeeding on liver health may differ between the sexes.

To address this knowledge gap, we used UK Biobank data to evaluate, separately in men and women, whether being breastfed in infancy is associated with MRI‑defined MASLD and MASH, as well as with incident cirrhosis, hepatocellular carcinoma, and liver‑related mortality. We further performed mediation analyses to explore potential biological pathways underlying these associations. This is the first large‑scale investigation to assess the sex‑dimorphic association between breastfeeding in infancy and risk of liver disease in adulthood.

## Methods

### Study design and participants

Details of the UK Biobank study design and population have been described previously [18]. Briefly, between 2006 and 2010, the study recruited around 0.5 million participants (aged 37–73) from the general population across 22 assessment centers in England, Scotland, and Wales. During baseline assessment, participants completed a touch-screen questionnaire, a standard interview, a series of physical measurements, and provided biological samples. Participants were followed through linkage with National Health Service records, with clinical outcomes coded using the WHO International Classification of Diseases (ICD) criteria.

Of the 501,965 participants, we sequentially excluded individuals who had withdrawn from the study (*n* = 1,271), those with missing or non‑definitive data on breastfeeding in infancy (including responses of “don’t know” or “prefer not to answer”) (*n* = 118,413), and those with a history of cirrhosis, HCC, other liver diseases, or alcohol/drug use disorders at or before baseline (*n* = 3,579) (Supplementary Tables 1 and 2). At last, 378,702 participants were included in the longitudinal analysis for incident cirrhosis, HCC, and liver‑related mortality (Supplementary Fig. 1). Additional, 26,850 participants with available multiparametric MRI data were included in the cross‑sectional, MRI‑based analysis of hepatic steatosis and inflammation.

### Assessment of exposure

Being breastfed in infancy was self-reported at baseline in response to the question “Were you breastfed when you were a baby?” and could select their answer from the choices including “Yes”, “No”, “Don’t know”, and “Prefer not to answer”.

### Assessment of outcome

In participants with available multiparametric MRI data, we used two validated MRI‑based biomarkers, liver proton density fat fraction (PDFF) for fat content and iron-corrected T1 (cT1) mapping for fibroinflammatory activity [19]. The liver MRI acquisition and analysis protocols have been previously described in detail [20]. In brief, liver MRI scans were acquired from a single transverse slice at the porta hepatis using a Siemens 1.5 T MAGNETOM Aera scanner (Siemens AG, Munich, Germany). Body composition was analyzed using AMRA Profiler Research (AMRA Medical AB, Linköping, Sweden) [21]. Liver PDFF was quantified by averaging nine strategically placed regions of interest, which excluded any inhomogeneities, major vessels, and bile ducts. Furthermore, a shortened modified look locker inversion was used to quantify liver T1. The derived liver T1 values were subsequently corrected for iron interference effects to generate the cT1 metric (expressed in milliseconds, ms). Based on MRI data, MASLD was defined as PDFF > 5.5%, and MASH as PDFF > 5.5% combined with cT1 value > 800 ms [19, 22, 23]. 

In the longitudinal analysis, incident cirrhosis, HCC, and liver‑related mortality were identified through linkage to National Health Service records, including hospital inpatient admissions and death registries. Further details can be found at https://www.ukbiobank.ac.uk/. Detailed information on the ICD-9/ICD-10 code used to define the outcomes is provided in Supplementary Table 2. For this study, hospital inpatient data were current through 31 October 2022 (England), 31 August 2022 (Scotland), and 31 May 2022 (Wales). Mortality data were available until 31 December 2023. Follow‑up was censored on these respective dates. Person‑years of follow‑up were calculated from the date of baseline assessment until the occurrence of an outcome event, death, or the end of follow‑up, whichever came first.

### Covariates

Demographic variables (age, sex, and ethnicity), socioeconomic indicators (education, household income, and Townsend Deprivation Index) [24], lifestyle habits (smoking status, alcohol consumption, physical activity, and dietary intake) were assessed by the baseline questionnaire. Ethnicity was categorized into white and others (South Asian, black, Chinese, and mixed ethnic background). Education levels were classified as follows: high (college/university degree or above), intermediate (advanced/advanced subsidiary levels, ordinary levels, general certificate of secondary education, certificate of secondary education, national vocational qualification or higher national diploma, or equivalent, and other professional qualifications), and low (none of the above). The average total household income was categorized into brackets: low (less than £18,000), median (£18,000 to £51,999), and high (greater than £52,000). Smoking status was self-reported and categorized as never, former, or current smoker. Alcohol intake (g/week) was calculated based on the average alcohol content by volume of each alcoholic beverage and the amount consumed per week. Physical activity was assessed using the International Physical Activity Questionnaire [25]. We defined a healthy diet as meeting ≥ 4 of 7 criteria: fruit (≥ 3 servings/day), vegetables (≥ 3 servings/day), fish (≥ 2 servings/week), whole grains (≥ 3 servings/day), refined grains (≤ 2 servings/day), processed meats (≤ 1 serving/week), and unprocessed meats (≤ 2 servings/week) [26]. Diabetes was identified based on self-reported physician diagnosis. Hypertension was defined as a systolic blood pressure ≥ 140 mmHg and/or a diastolic blood pressure ≥ 90 mmHg, self-reported hypertension, or antihypertensive medication use.

### Statistical analysis

Baseline characteristics are presented as counts (percentages) for categorical variables and as means (standard deviations) for continuous variables. Group imbalance was assessed using standardized mean differences (SMDs) and considered an SMD greater than 0.1 as a meaningful difference. To reduce potential inferential bias and to enhance statistical power, we employed multiple imputations with chained equations for any missing covariate data, ensuring that participants with incomplete data were not excluded from our analyses [27]. Further details on the number of missing covariates can be found in the Supplementary Table 3.

All analyses were stratified by sex, and sex differences were formally tested using interaction terms where applicable. We employed logistic regression to examine the association between breastfeeding in infancy and MRI-defined MASLD/MASH in adulthood. For the longitudinal analysis of incident cirrhosis, HCC, and liver-related mortality, Cox proportional hazards models were used. In both sets of analyses, three sequentially adjusted models were applied. Model 1 was adjusted for baseline age and ethnicity. Model 2 incorporated several variables, including income, education, and the Townsend deprivation index. Model 3 was further adjusted for BMI, smoking status, alcohol intake, physical activity, healthy diet, maternal smoking around birth, and history of diabetes and hypertension, in addition to all variables in Model 2. Results from the logistic regression are presented as odds ratios (ORs), and those from the Cox models as hazard ratios (HRs), each with its corresponding 95% confidence intervals (CIs).

To investigate the mediating pathways through which infant breastfeeding influences offspring liver health and to examine potential sex differences in these mechanisms, we performed a mediation analysis within the counterfactual framework for all serum biochemistry parameters. The analysis aims to decompose the total effect of breastfeeding on severe liver disease, defined as a composite endpoint of cirrhosis, HCC, and liver‑related mortality, into natural direct effects and natural indirect effects. Potential nonlinear relationships between the mediators and the outcome were accommodated by modeling the mediators using restricted cubic splines with four knots in the outcome model. All analyses were conducted separately for male and female offspring. For each mediator, two models were specified: a linear regression model for the mediator on breastfeeding status and covariates, and a Cox proportional hazards model for the composite endpoint on breastfeeding status, the mediator (modeled with splines), and covariates. Both models adjusted for the same comprehensive set of covariates used in the primary analysis (Model 3), including age, ethnicity, income, education, Townsend deprivation index, body mass index, smoking status, alcohol intake, physical activity, healthy diet, maternal smoking around birth, diabetes, and hypertension. Natural direct and indirect effects were estimated via the product method with 1,000 bootstrap resamples. Sex differences in the mediating pathways were formally tested by comparing the natural indirect effect estimates between males and females, with the false discovery rate (FDR) method (Benjamini-Hochberg) applied to correct for multiple testing.

The potential interaction effects were evaluated using likelihood ratio tests by comparing models with and without relevant multiplicative interaction terms. To assess the robustness of the primary findings, we conducted sensitivity analyses that (1) restricted the analysis to participants with complete covariate data and (2) excluded participants who developed outcomes or died within the first two years of follow-up. Furthermore, mortality from accidents (ICD-10 codes V01-X59) was selected as a negative control outcome to evaluate potential unmeasured confounding, as it is unlikely to be causally influenced by breastfeeding in infancy [28].

All analyses were conducted using R software, version 4.3.2 (R Development Core Team, Vienna, Austria). Statistical significance was defined as a two-tailed P value < 0.05.

## Result

### Breastfeeding and MRI-derived PDFF and cT1

Of the 26,850 participants with multiparametric MRI data, 14,781 were female, with a mean age of 54.3 years. A total of 8,857 (74.5%) male and 10,645 (71.2%) female participants reported being breastfed in infancy. The characteristics of the 26,850 participants who underwent liver MRI are presented in Table 1, stratified by sex and breastfeeding status in infancy. Within this population, individuals who were breastfed in infancy were, on average, slightly older, more likely to be of non-White ethnicity, and had mothers who reported a lower prevalence of smoking around birth, compared to those who were not breastfed. SMDs for most covariates were below 0.1 in both males and females, indicating a generally comparable distribution of baseline characteristics between the exposure groups.

**Table 1:** Characteristics of individuals with available liver MRI data, by sex and breastfeeding status in infancy

	Males			Females		
	Being breastfed in infancy			Being breastfed in infancy		
**Characteristics**	Yes (*n* = 8857)	No (*n* = 3032)	SMD	Yes (*n* = 10645)	No (*n* = 4316)	SMD
Age, mean (SD), y	55.79 (7.41)	52.36 (7.62)	0.457	54.78 (7.18)	51.69 (7.32)	0.427
Townsend Deprivation Index, mean (SD)	−1.97 (2.71)	−1.83 (2.73)	0.051	−1.87 (2.72)	−1.81 (2.78)	0.021
White ethnicity, n (%)	8231 (92.9)	2900 (95.6)	0.117	9810 (92.2)	4094 (94.9)	0.110
BMI (kg/m^2^), mean (SD)	26.99 (3.62)	27.15 (3.78)	0.043	25.93 (4.44)	25.96 (4.59)	0.007
Education level, n (%)			0.105			0.062
Low	520 (5.9)	146 (4.8)		542 (5.1)	244 (5.7)	
Intermediate	3709 (41.9)	1423 (46.9)		4976 (46.7)	2124 (49.2)	
High	4628 (52.3)	1463 (48.3)		5127 (48.2)	1948 (45.1)	
Average total household income, n (%)			0.019			0.068
Low	827 (9.3)	278 (9.2)		1384 (13.0)	540 (12.5)	
Median	4326 (48.8)	1457 (48.1)		5676 (53.3)	2182 (50.6)	
High	3704 (41.8)	1297 (42.8)		3585 (33.7)	1594 (36.9)	
Smoking status, n (%)			0.107			0.08
Never	5036 (56.9)	1856 (61.2)		6863 (64.5)	2883 (66.8)	
Former	3221 (36.4)	949 (31.3)		3286 (30.9)	1190 (27.6)	
Current	600 (6.8)	227 (7.5)		496 (4.7)	243 (5.6)	
Alcohol intake, g/week	158.89 (145.68)	150.93 (141.94)	0.055	81.29 (88.14)	82.15 (88.79)	0.010
Physical activity, n (%)			0.025			0.042
Low	1604 (18.1)	578 (19.1)		1835 (17.2)	813 (18.8)	
Moderate	3593 (40.6)	1215 (40.1)		4674 (43.9)	1852 (42.9)	
High	3660 (41.3)	1239 (40.9)		4136 (38.9)	1651 (38.3)	
Healthy diet, n (%)	4958 (56.0)	1577 (52.0)	0.080	7396 (69.5)	2839 (65.8)	0.079
Diabetes, n (%)	318 (3.6)	90 (3.0)	0.035	197 (1.9)	57 (1.3)	0.042
Hypertension, n (%)	4916 (55.5)	1556 (51.3)	0.084	4056 (38.1)	1424 (33.0)	0.107
Maternal smoking around birth, n (%)	2429 (27.4)	1045 (34.5)	0.153	2751 (25.8)	1502 (34.8)	0.196
ALT, mean (SD), U/L	26.55 (14.66)	28.05 (15.40)	0.099	19.33 (11.14)	19.06 (10.58)	0.024
AST, mean (SD), U/L	27.63 (11.26)	27.92 (11.86)	0.025	23.93 (8.03)	23.45 (7.17)	0.063
TC, mean (SD), mmol/L	5.60 (1.07)	5.62 (1.07)	0.017	5.87 (1.09)	5.74 (1.03)	0.122
LDL-C, mean (SD), mmol/L	3.57 (0.83)	3.59 (0.83)	0.018	3.61 (0.84)	3.53 (0.80)	0.097
HDL-C, mean (SD), mmol/L	1.31 (0.30)	1.30 (0.30)	0.057	1.64 (0.37)	1.61 (0.36)	0.083
HbA1c, mean (SD), mmol/mol	35.38 (5.69)	34.92 (5.88)	0.080	34.94 (4.76)	34.27 (4.50)	0.144
FBG, mean (SD), mmol/L	5.05 (1.07)	5.02 (1.07)	0.025	4.97 (0.86)	4.92 (0.86)	0.056
TG, mean (SD), mmol/L	1.87 (1.04)	1.95 (1.13)	0.073	1.42 (0.78)	1.39 (0.76)	0.046

MRI, magnetic resonance imaging; SMD, standardized mean difference; SD, standard deviation; BMI, body mass index; ALT, alanine aminotransferase; AST, aspartate transaminase; TC, total cholesterol; HDL-C, high-density lipoprotein cholesterol; LDL-C, low-density lipoprotein cholesterol; FBG, fasting blood glucose; TG, triglycerides; HbA1c, glycated hemoglobin

Based on liver PDFF and cT1 value, 6,274 (23.4%) and 1,161 (4.3%) participants were defined as MASLD and MASH, respectively. Table 2 shows the associations of breastfeeding in infancy with outcomes defined by MRI. For MRI-defined MASLD, although a protective association was observed in females (adjusted OR, 0.89; 95% CI, 0.80–0.99) with no significant association in males, the interaction by sex was not statistically significant (P for interaction = 0.301). For MRI-defined MASH, breastfeeding was associated with a reduced risk exclusively in females (adjusted OR, 0.79; 95% CI, 0.65–0.97), with a significant sex interaction (P for interaction = 0.023).

**Table 2:** Associations of breastfeeding in infancy with MRI-defined outcomes

	Males		Females		*P* for interaction
	Being breastfed in infancy		Being breastfed in infancy		
	No	Yes	No	Yes	
MASLD by PDFF > 5.5%					
Events No./total No.	961/3032	2684/8857	811/4316	1818/10,645	
Model 1^1^ OR (95% CI)	1 (Reference)	0.97 (0.89–1.06)	1 (Reference)	0.86 (0.78–0.94)	0.430
Model 2^1,2^ OR (95% CI)	1 (Reference)	0.99 (0.90–1.09)	1 (Reference)	0.88 (0.80–0.97)	0.396
Model 3^1,2,3^ OR (95% CI)	1 (Reference)	1.03 (0.94–1.13)	1 (Reference)	0.89 (0.80–0.99)	0.301
MASH by PDFF > 5.5% and cT1 > 800ms					
Events No./total No.	181/3032	476/8857	183/4316	321/10,645	
Model 1^1^ OR (95% CI)	1 (Reference)	0.97 (0.81–1.16)	1 (Reference)	0.74 (0.62–0.90)	0.056
Model 2^1,2^ OR (95% CI)	1 (Reference)	0.99 (0.83–1.20)	1 (Reference)	0.77 (0.64–0.93)	0.048
Model 3^1,2,3^ OR (95% CI)	1 (Reference)	1.08 (0.90–1.31)	1 (Reference)	0.79 (0.65–0.97)	0.023

MASH, metabolic dysfunction-associated steatohepatitis; OR, odds ratio; CI, confidence interval; PDFF, proton density fat fraction; cT1, iron-corrected T1 mapping

^1^Adjusted for age and ethnicity

^2^Adjusted for income, education, and Townsend deprivation index

^3^Adjusted for body mass index, smoking status, alcohol intake, physical activity, healthy diet, maternal smoking around birth, history of diabetes and hypertension

### Breastfeeding and adverse liver outcomes

The longitudinal analyses included 378,702 participants with a mean age of 55.8 years, of whom 218,488 (57.7%) were female. At baseline, a total of 120,935 (75.5%) male participants and 153,109 (70.1%) female participants reported being breastfed in infancy. The baseline characteristics are shown in Supplementary Table 4.

**Fig. 1:**
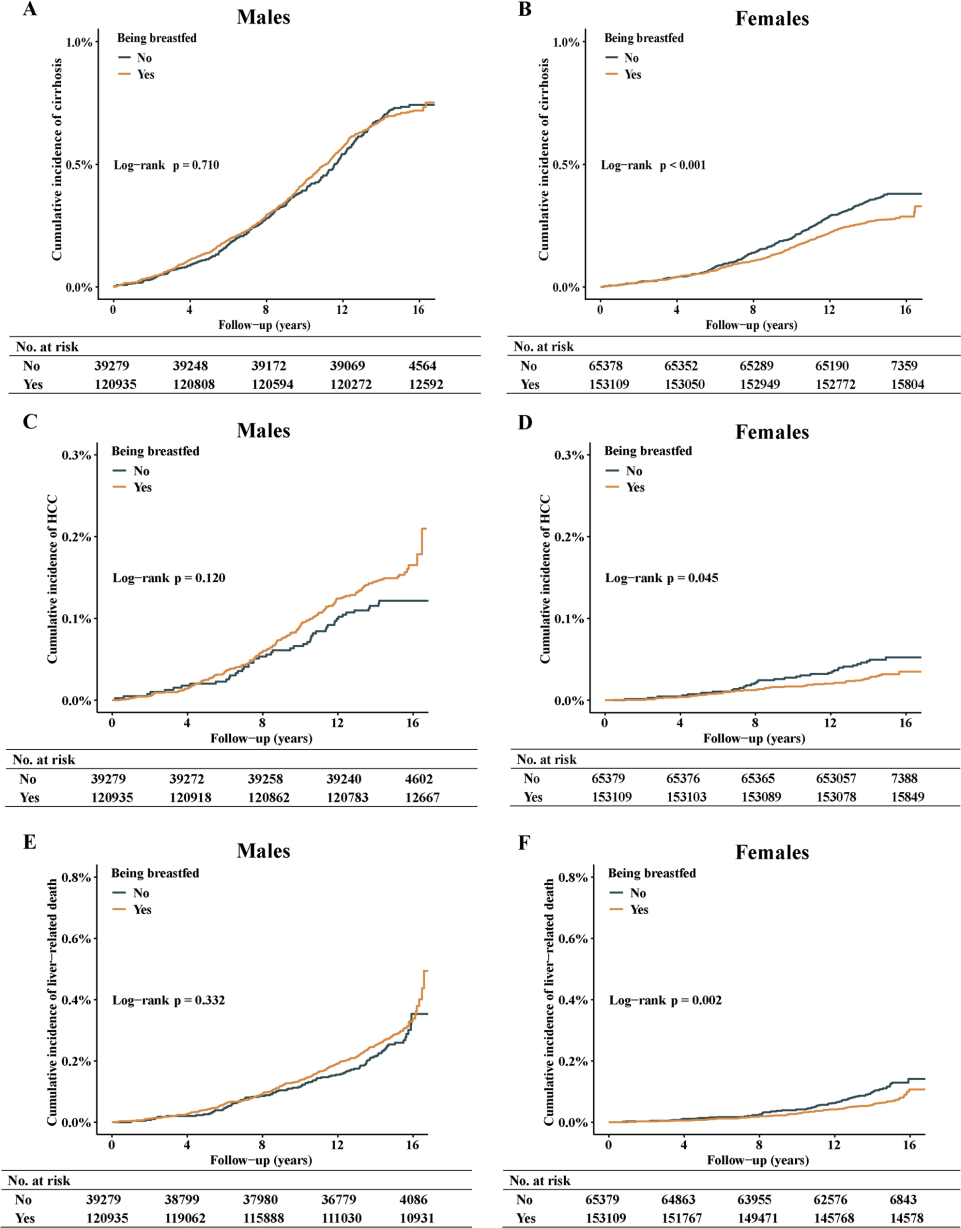
Cumulative incidence of cirrhosis, HCC, and liver-related mortality according to breastfeeding status in infancy, stratified by sex. **(A)** Cirrhosis in males; **(B)** Cirrhosis in females; **(C)** HCC in males; **(D)** HCC in females; **(E)** Liver-related mortality in males; **(F)** Liver-related mortality in females. HCC, hepatocellular carcinoma

Over a median (interquartile range) follow-up of 14.9 (1.4) years, a total of 1,750 cases of cirrhosis (659 females), 320 cases of HCC (82 females), and 610 liver-related deaths (181 females) were identified. Kaplan-Meier analysis revealed significant sex-specific differences in the long-term hepatic benefits of breastfeeding. In females, being breastfed in infancy correlated with a significantly lower cumulative incidence of cirrhosis, hepatocellular carcinoma (HCC), and liver-related mortality (log-rank test, *P* < 0.001, *P* = 0.045, and *P* = 0.002, respectively; Fig. 1). These associations were further supported by multivariable Cox regression analyses, which revealed risk reductions of 28% for cirrhosis (adjusted HR = 0.71, 95% confidence interval [CI]: 0.60–0.83), 48% for HCC (adjusted HR = 0.52, 95% CI: 0.33–0.82), and 37% for liver-related mortality (adjusted HR = 0.63, 95% CI: 0.46–0.86) among women who had been breastfed (Table 3). In contrast, among males, no significant associations were observed, with cumulative incidence curves overlapping (all log-rank *P* > 0.05; Fig. 1) and adjusted risks showing no significant protective effect for cirrhosis (adjusted HR, 0.90; 95% CI, 0.78–1.04; P for interaction = 0.047), HCC (adjusted HR, 0.99; 95% CI, 0.71–1.37; P for interaction = 0.030), or liver-related mortality (adjusted HR, 1.05; 95% CI, 0.83–1.32; P for interaction = 0.008; Table 3).

**Table 3:** Associations of breastfeeding in infancy with cirrhosis, HCC, and liver-related mortality in adulthood

	Males		Females		*P* for interaction
	Being breastfed in infancy		Being breastfed in infancy		
	No	Yes	No	Yes	
Cirrhosis					
Events No./total No.	275/39,279	816/120,935	240/65,379	419/153,009	
Model 1^1^ HR (95% CI)	1 (Reference)	0.83 (0.72–0.96)	1 (Reference)	0.65 (0.56–0.77)	0.060
Model 2^1,2^ HR (95% CI)	1 (Reference)	0.89 (0.77–1.02)	1 (Reference)	0.69 (0.59–0.81)	0.041
Model 3^1,2,3^ HR (95% CI)	1 (Reference)	0.90 (0.78–1.04)	1 (Reference)	0.71 (0.60–0.83)	0.047
HCC					
Events No./total No.	47/39,279	191/120,935	33/65,379	49/153,009	
Model 1^1^ HR (95% CI)	1 (Reference)	0.98 (0.71–1.35)	1 (Reference)	0.49 (0.32–0.77)	0.021
Model 2^1,2^ HR (95% CI)	1 (Reference)	1.01 (0.73–1.40)	1 (Reference)	0.51 (0.33–0.80)	0.020
Model 3^1,2,3^ HR (95% CI)	1 (Reference)	0.99 (0.71–1.37)	1 (Reference)	0.52 (0.33–0.82)	0.030
Liver-related mortality					
Events No./total No.	98/39,279	331/120,935	75/65,379	106/153,009	
Model 1^1^ HR (95% CI)	1 (Reference)	0.96 (0.76–1.20)	1 (Reference)	0.55 (0.41–0.75)	0.003
Model 2^1,2^ HR (95% CI)	1 (Reference)	1.04 (0.82–1.31)	1 (Reference)	0.61 (0.45–0.82)	0.003
Model 3^1,2,3^ HR (95% CI)	1 (Reference)	1.05 (0.83–1.32)	1 (Reference)	0.63 (0.46–0.86)	0.008

HCC, hepatocellular carcinoma; HR, hazard ratio; CI, confidence interval

^1^Adjusted for age and ethnicity

^2^Adjusted for income, education, and Townsend deprivation index

^3^Adjusted for body mass index, smoking status, alcohol intake, physical activity, healthy diet, maternal smoking around birth, history of diabetes and hypertension

### Mediation analysis

To investigate the biological pathways through which breastfeeding in infancy may protect female offspring against severe liver disease (cirrhosis, HCC, or liver-related mortality), we conducted a sex-stratified mediation analysis using a counterfactual framework. Multiple biochemical indicators were examined, and insulin-like growth factor 1 (IGF-1) was the only biomarker that demonstrated a significant sex‑specific mediating effect. Specifically, a protective natural indirect effect was observed in women (β =−0.027; 95% CI: −0.040 to −0.014) but not in men (β = 0.014; 95% CI: −0.006 to 0.031), with a statistically significant difference between sexes (FDR < 0.001; Supplementary Tables 5 & 6).

In further detail, breastfeeding in infancy was associated with higher serum IGF-1 levels in females (β = 0.10; 95% CI: 0.05 to 0.16) but not in males (β = −0.05; 95% CI: −0.12 to 0.01; P for interaction = 0.039; Supplementary Table 7). Furthermore, in both sexes, the association between IGF-1 and severe liver disease followed an L-shaped pattern (Supplementary Table 8).

### Sensitivity analysis

In sensitivity analyses, excluding either participants with missing covariate data (Supplementary Table 9) or early-onset cases within the first 2 years (Supplementary Table 10), the results were similar to the main analysis. As expected for the negative control outcome, breastfeeding in infancy showed no association with accidental mortality in either males or females, with no significant interaction by sex (Supplementary Table 11).

## Discussion

To the best of our knowledge, this is the first large-scale study to demonstrate that being breastfed in infancy is associated with a lower risk of developing MASH, cirrhosis, HCC, and liver-related death in adulthood, specifically among women.

Epidemiological evidence on the association between breastfeeding in infancy and the risk of metabolic liver disease later in life remains scarce and confined to pediatric populations. Earlier case-control studies have linked breastfeeding to a lower incidence of NAFLD in adolescents, as well as a reduced risk of pediatric NASH and fibrosis [29, 30]. Nevertheless, it is unclear whether such protection extends into adulthood and whether it differs by sex. In our study, breastfeeding in infancy was associated with a reduced risk of MRI-defined MASLD among women but not among men, although the sex interaction was not statistically significant (P for interaction = 0.301). Notably, a reduced risk of MRI‑defined MASH was observed only in females, with a significant sex interaction (P for interaction = 0.023). This female-specific protective pattern is consistent with prior epidemiological observations. For example, exposure to early-life famine has been associated with an elevated risk of adult fatty liver disease predominantly in women, suggesting a particular sensitivity of the female liver to early nutritional programming [13]. Moreover, as expected, breastfeeding in infancy was also associated with a lower risk of cirrhosis, HCC, and liver-related mortality in females, but not in males, further supporting a long-term, sex-specific hepatic benefit of breastfeeding in infancy.

The observed female-specific protective effects are biologically plausible. Previous research showed that breast milk-derived betaine transiently increases the abundance of Akkermansia spp. in the early-life gut, a change that can lead to sustained metabolic improvements, demonstrating the durable impact of early dietary exposures [31]. In animal models, maternal intake of cinnamon-derived bioactive compounds during lactation selectively improves long-term insulin sensitivity specifically in female offspring [14]. Thus, it is likely that specific bioactive factors in breast milk act in a sex‑specific manner to improve metabolic regulation, thereby attenuating the long‑term development of MASH and its associated complications.

Human breast milk plays a critical role in shaping the early-life gut microbiota, supporting intestinal barrier function, and guiding immune system development [32, 33]. Importantly, it has been shown that supplementation with human milk oligosaccharides beginning at weaning and maintained for 8 weeks led to durable, female-specific improvements in gut barrier function, including sustained reductions in intestinal permeability and increased expression of tight junction proteins [34]. Given the key role of a disrupted gut-liver axis in hepatic inflammation [35–38], breastfeeding may promote sustained reinforcement of intestinal barrier integrity and a balanced immune environment, and thereby prevent MASH and adverse liver outcome in a sex‑specific manner.

Our mediation analysis further elucidates the potential pathways linking early breastfeeding to long‑term liver outcomes. IGF‑1 is a growth factor with anti‑inflammatory properties that attenuate MASLD progression [17]. Intriguingly, our analysis revealed that the protective association between breastfeeding in infancy and severe liver disease was significantly mediated by IGF‑1 in females, but not in males. This observation aligns with human evidence that early-life nutritional stress durably alters IGF-1 profiles in women [15], and animal studies demonstrating that the metabolic benefits of growth hormone (GH)/IGF-1 axis modulation are evident primarily in females [16]. Collectively, the sex‑specific hepatic benefit of breastfeeding in women may be partly attributable to its sustained, positive modulation of IGF‑1 signaling.

### Strengths and limitations

Our study has several strengths. First, by combining MRI phenotyping with prospective follow-up data, this large-scale study offers the first evidence that breastfeeding in infancy is linked to a broad spectrum of adult liver diseases, from subclinical MASLD/MASH to incident cirrhosis, HCC, and liver-related mortality. Second, the study benefited from detailed information on socioeconomic status, lifestyle factors, comorbid conditions, and other relevant covariates, enabling rigorous adjustment for potential confounders. Nonetheless, several limitations warrant consideration. First, observational design precludes causal inference, although the consistency of results after excluding early-onset cases supports robustness. Second, exposure assessment relied on retrospective self-report of breastfeeding in infancy decades later. Although such recall is subject to misclassification, the non‑differential nature likely attenuates the observed associations. Reassuringly, our negative control analysis found no association between breastfeeding and accidental mortality, an outcome with no plausible biological link to breastfeeding in infancy. This finding helps rule out major differential recall bias and suggests that unmeasured familial or socioeconomic factors are unlikely to account for the observed associations. Finally, as our study population was predominantly of European ancestry, the generalizability of our findings to other racial and ethnic groups requires further validation.

## Conclusions

This study demonstrates that breastfeeding in infancy is associated with a reduced risk of MASH, cirrhosis, HCC, and liver-related mortality in women, with no comparable association in men. The effect was partially mediated by circulating IGF-1, suggesting a potential mechanistic pathway. These results point to a long-term, female-specific liver benefit conferred by breastfeeding in infancy and highlight its potential importance in liver disease prevention.

## Supplementary Information

Supplementary Material 1

## Data Availability

All UK Biobank information is available online (https://www.ukbiobank.ac.uk/), and data can be accessed upon application (https://www.ukbiobank.ac.uk/enable-your-research/apply-for-access).

## Abbreviations

MASLD: Metabolic dysfunction-associated steatotic liver disease
MASH: Metabolic dysfunction-associated steatohepatitis
HCC: Hepatocellular carcinoma
PDFF: Proton density fat fraction
cT1: Iron-corrected T1 mapping
NASH: Nonalcoholic steatohepatitis
SMD: Standardized mean difference
SD: Standard deviation
BMI: Body mass index
ALT: Alanine aminotransferase
AST: Aspartate transaminase
TC: Total cholesterol
HDL-C: High-density lipoprotein cholesterol
LDL-C: Low-density lipoprotein cholesterol
FBG: Fasting blood glucose
TG: Triglycerides
HbA1c: Glycated hemoglobin
