# Supplementary material for: Breastfeeding in infancy confers sex-specific, long-term protection against metabolic dysfunction-associated steatohepatitis and adverse liver outcomes
Source: Biol Sex Differ. 2026 May 12;17:127. doi: 10.1186/s13293-026-00919-4 (PMC13335326; doi:10.1186/s13293-026-00919-4)
Supplement: Supplementary file 1 — Supplementary Material 1 [file 13293_2026_919_MOESM1_ESM.docx]

**Supplemental Online Content**

**Supplementary Figure. 1. Flow Chart**

**Supplementary Table 1. ICD codes for diseases used as exclusion criteria**

**Supplementary Table 2. ICD codes utilized for defining outcomes**

**Supplementary Table 3. The numbers (percentages) of participants with missing covariates**

**Supplementary Table 4. Baseline characteristics of the study population according to breastfeeding status in infancy**

**Supplementary Table 5. Sex differences in natural indirect effects of candidate mediators**

**Supplementary Table 6. Sex differences in natural direct effects of candidate mediators**

**Supplementary Table 7. Association between breastfeeding in infancy and serum IGF-1 levels by sex**

**Supplementary Table 8. Association between serum levels of IGF-1 and severe liver disease by sex**

**Supplementary Table 9. Association of breastfeeding in infancy with MASLD, MASH, cirrhosis, HCC, and liver-related mortality in adulthood when excluding participants with missing covariates**

**Supplementary Table 10. Association of breastfeeding in infancy with incident MASLD, MASH, cirrhosis, HCC, and liver-related mortality in adulthood using landmark 2-year analysis**

**Supplementary Table 11. Association of breastfeeding in infancy with accident death**

UK biobank participants, n=501,965

Exclusion:

- People lost to follow-up, n=1,271
- People with incomplete data for exposures, n=118,413
- People with cirrhosis, HCC, other liver diseases, or alcohol/drug abuse at/before baseline, n=3,579

Participants included in the longitudinal analysis, n=378,702

Exclusion:

- People with missing data for PDFF or cT1 value, n=351,866

Participants included in MRI‑based analysis, n=26,850

**Supplementary Figure 1. Flow Chart.** Abbreviations: MASLD, metabolic dysfunction-associated steatotic liver disease; HCC, hepatocellular carcinoma; MASH, metabolic dysfunction-associated steatohepatitis; PDFF, proton density fat fraction; cT1, iron-corrected T1 mapping.

**Supplementary Table 1. ICD codes for diseases used as exclusion criteria**

| **Disease at or before baseline** | **ICD-10 codes** | **ICD-9 codes** |
| --- | --- | --- |
| Alcoholic liver disease | K70 | 571.0, 571.1, 571.2, 571.3 |
| Viral hepatitis | B16, B17, B18, B19 | 070 |
| Autoimmune liver disease | K83.0, K74.3, K75.4 | 576.1 |
| Hemochromatosis | E83.1 | 275.0 |
| Wilson | E83.0 | 275.1 |
| Alpha-1-antitrypsin deficiency | E88.0 | 273.4 |
| Budd-Chiari | I82.0, K76.5 | 453.0 |
| Chronic hepatitis, unspecified | K73.9, K73.2 | 571.4 |
| Secondary or unspecified biliary cirrhosis | K74.4, K74.5 | 571.6 |
| Drug use disorder | F11, F12, F13, F14, F16, F18, F19 | 304, 305 |
| Liver transplantation | Z94.4 | V42.7 |
|  |  |  |
| Alcohol use disorder | E24.4, G62.1, I42.6, K29.2, G31.2, G72.1, K85.2, K86.0, T51.0, T51.9, Y57.3, X65, Z50.2, Z71.4, Z72.1 | 357.5, 425.5, 535.3, 331.7, 359.4, 980.0, 980.9, E939.3, V69.2 |
| MASLD | K76.0, K75.8 | 571.8 |

Abbreviations: MASLD, metabolic dysfunction-associated steatotic liver disease.

**Supplementary Table 2. ICD codes utilized for defining outcomes**

| **Outcomes** | **ICD-10 codes** | **ICD-9 codes** |
| --- | --- | --- |
| **Cirrhosis** |  |  |
| Hepatic sclerosis | K74.1 | 571.5 |
| Hepatic fibrosis with hepatic sclerosis | K74.2 | 571.5 |
| Other and unspecified cirrhosis of liver | K74.6 | 571.5 |
| Portal hypertension | K76.6 | 572.3 |
| Esophageal varices with bleeding | I85.0, I98.2 | 456.0 |
| Esophageal varices without bleeding | I85.9, I98.3 | 456.1 |
| Hepatorenal syndrome | K76.7 | 572.4 |
| **HCC** | C22.0 | 155.2 |
| **Liver-related mortality** |  |  |
| Alcoholic liver disease | K70 | - |
| Toxic liver disease | K71 | - |
| Hepatic failure, not elsewhere classified | K72 | - |
| Chronic hepatitis, not elsewhere classified | K73 | - |
| Fibrosis and cirrhosis of liver | K74 | - |
| Other inflammatory liver diseases | K75 | - |
| Other diseases of liver | K76 | - |
| Liver disorders in diseases classified elsewhere | K77 | - |
| Oesophageal varices | I85 | - |
| HCC | C22.0 | - |

Abbreviations: HCC, hepatocellular carcinoma.

**Supplementary Table 3. Number and percentages of participants with missing data for each covariate**

| Covariates | MRI‑based analysis | | Longitudinal analysis | |
| --- | --- | --- | --- | --- |
|  | N* | Missing rate (%) | N* | Missing rate (%) |
| Physical activity | 4282 | 15.9% | 82024 | 21.7% |
| Average total household income | 2413 | 9.0% | 53017 | 14.0% |
| Maternal smoking around birth | 2572 | 9.6% | 40263 | 10.6% |
| Education | 424 | 1.6% | 6258 | 1.7% |
| Alcohol intake | 145 | 0.5% | 4456 | 1.2% |
| BMI | 40 | 0.1% | 1937 | 0.5% |
| Smoking status | 39 | 0.1% | 1227 | 0.3% |
| Ethnicity | 60 | 0.2% | 1152 | 0.3% |
| Diabetes | 35 | 0.1% | 1049 | 0.3% |
| Townsend Deprivation Index | 21 | 0.1% | 468 | 0.1% |

*N represents the number of missing responses. Abbreviations: BMI, body mass index.

**Supplementary Table 4. Baseline characteristics of the study population according to breastfeeding status in infancy**

|  | **Males** | | | **Females** | | |
| --- | --- | --- | --- | --- | --- | --- |
|  | **Being breastfed in infancy** | | | **Being breastfed in infancy** | | |
| **Characteristics** | Yes (n=120935) | No (n=39279) | SMD | Yes (n=153109) | No (n=65379) | SMD |
| Age, mean (SD), y | 56.90 (7.99) | 53.07 (8.37) | 0.468 | 56.66 (7.75) | 53.61 (8.23) | 0.382 |
| Townsend Deprivation Index, mean (SD) | -1.35 (3.11) | -1.19 (3.15) | 0.051 | -1.39 (3.01) | -1.30 (3.05) | 0.029 |
| White ethnicity, n (%) | 107582 (89.0) | 37486 (95.4) | 0.243 | 135725 (88.6) | 61753 (94.5) | 0.210 |
| BMI (kg/m^2^), mean (SD) | 27.81 (4.19) | 27.88 (4.35) | 0.015 | 26.99 (5.13) | 27.06 (5.35) | 0.013 |
| Education level, n (%) |  |  | 0.097 |  |  | 0.083 |
| Low | 18185 (15.0) | 6058 (15.4) |  | 22640 (14.8) | 10197 (15.6) |  |
| Intermediate | 57118 (47.2) | 20191 (51.4) |  | 77254 (50.5) | 34992 (53.5) |  |
| High | 45632 (37.7) | 13030 (33.2) |  | 53215 (34.8) | 20190 (30.9) |  |
| Average total household income, n (%) |  |  | 0.047 |  |  | 0.042 |
| Low | 24221 (20.0) | 7144 (18.2) |  | 38269 (25.0) | 15367 (23.5) |  |
| Median | 60807 (50.3) | 20280 (51.6) |  | 79083 (51.7) | 33815 (51.7) |  |
| High | 35907 (29.7) | 11855 (30.2) |  | 35757 (23.4) | 16197 (24.8) |  |
| Smoking status, n (%) |  |  | 0.095 |  |  | 0.070 |
| Never | 59776 (49.4) | 20694 (52.7) |  | 92136 (60.2) | 39451 (60.3) |  |
| Former | 46901 (38.8) | 13448 (34.2) |  | 48561 (31.7) | 19439 (29.7) |  |
| Current | 14258 (11.8) | 5137 (13.1) |  | 12412 ( 8.1) | 6489 ( 9.9) |  |
| Alcohol intake, g/week | 157.75 (164.24) | 159.30 (170.54) | 0.010 | 71.50 (91.04) | 74.74 (95.16) | 0.035 |
| Physical activity, n (%) |  |  | 0.020 |  |  | 0.030 |
| Low | 22256 (18.4) | 7299 (18.6) |  | 22640 (14.8) | 10197 (15.6) |  |
| Moderate | 46890 (38.8) | 14840 (37.8) |  | 77254 (50.5) | 34992 (53.5) |  |
| High | 51789 (42.8) | 17140 (43.6) |  | 53215 (34.8) | 20190 (30.9) |  |
| Healthy diet, n (%) | 64998 (53.7) | 19076 (48.6) | 0.104 | 104874 (68.5) | 41584 (63.6) | 0.103 |
| Diabetes, n (%) | 8506 (7.0) | 2091 (5.3) | 0.071 | 5840 ( 3.8) | 2204 ( 3.4) | 0.025 |
| Hypertension, n (%) | 75524 (62.5) | 22800 (58.0) | 0.090 | 74771 (48.8) | 28724 (43.9) | 0.098 |
| Maternal smoking around birth, n (%) | 32322 (26.8) | 13978 (35.6) | 0.192 | 38219 (25.0) | 22530 (34.5) | 0.209 |
| ALT, mean (SD), U/L | 27.09 (15.02) | 28.43 (15.63) | 0.088 | 20.17 (11.98) | 20.12 (12.09) | 0.003 |
| AST, mean (SD), U/L | 28.02 (10.98) | 28.31 (11.16) | 0.026 | 24.50 (9.27) | 24.12 (8.93) | 0.042 |
| TC, mean (SD), mmol/L | 5.50 (1.12) | 5.56 (1.11) | 0.054 | 5.88 (1.12) | 5.78 (1.11) | 0.084 |
| LDL-C, mean (SD), mmol/L | 3.49 (0.86) | 3.54 (0.85) | 0.062 | 3.63 (0.87) | 3.57 (0.86) | 0.069 |
| HDL-C, mean (SD), mmol/L | 1.29 (0.31) | 1.28 (0.31) | 0.041 | 1.60 (0.38) | 1.57 (0.37) | 0.058 |
| HbA1c, mean (SD), mmol/mol | 36.53 (7.56) | 35.79 (7.33) | 0.100 | 35.90 (5.99) | 35.21 (5.82) | 0.117 |
| FBG, mean (SD), mmol/L | 5.19 (1.40) | 5.12 (1.31) | 0.047 | 5.07 (1.07) | 5.03 (1.05) | 0.043 |
| TG, mean (SD), mmol/L | 1.96 (1.14) | 2.01 (1.18) | 0.045 | 1.55 (0.86) | 1.52 (0.87) | 0.027 |

Abbreviations: SMD, standardized mean difference; SD, standard deviation; BMI, body mass index; ALT, alanine aminotransferase; AST, aspartate transaminase; TC, total cholesterol; HDL-C, high-density lipoprotein cholesterol; LDL-C, low-density lipoprotein cholesterol; FBG, fasting blood glucose; TG, triglycerides; HbA1c, glycated hemoglobin.

**Supplementary Table 5. Sex differences in natural indirect effects of candidate mediators**

| Mediator | Males, β (95% CI) | Females, β (95% CI) | FDR for difference |
| --- | --- | --- | --- |
| IGF-1 | 0.014 (-0.006 to 0.031) | -0.027 (-0.040 to -0.014) | <0.001 |
| Alkaline phosphatase | -0.001 (-0.004 to 0.001) | 0.009 (0.003 to 0.018) | 0.203 |
| Albumin | 0.005 (-0.002 to 0.011) | -0.005 (-0.011 to 0.003) | 0.392 |
| Vitamin D | 0.002 (-0.004 to 0.013) | -0.009 (-0.017 to -0.002) | 0.392 |
| Total protein | 0.002 (-0.002 to 0.005) | -0.004 (-0.009 to 0.001) | 0.493 |
| Testosterone | 0.002 (-0.000 to 0.006) | 0.015 (0.003 to 0.030) | 0.464 |
| Calcium | 0.001 (-0.004 to 0.005) | -0.003 (-0.009 to -0.000) | 0.643 |
| Creatinine | -0.005 (-0.015 to 0.012) | -0.015 (-0.026 to -0.007) | 0.643 |
| Lipoprotein A | 0.003 (-0.016 to 0.018) | -0.020 (-0.060 to 0.005) | 0.643 |
| SHBG | -0.003 (-0.011 to 0.002) | -0.010 (-0.018 to -0.001) | 0.643 |
| Urea | 0.035 (0.020 to 0.049) | 0.022 (0.009 to 0.035) | 0.643 |
| CRP | -0.008 (-0.034 to 0.003) | -0.025 (-0.065 to -0.004) | 0.823 |
| HbA1c | -0.009 (-0.019 to -0.002) | -0.013 (-0.023 to -0.005) | 0.933 |
| Urate | -0.002 (-0.006 to 0.002) | 0.000 (-0.002 to 0.003) | 0.933 |
| Glucose | -0.001 (-0.009 to 0.005) | 0.003 (-0.006 to 0.009) | 0.893 |
| Triglycerides | 0.003 (-0.004 to 0.011) | -0.001 (-0.009 to 0.009) | 0.893 |
| Phosphate | -0.008 (-0.015 to -0.001) | -0.010 (-0.019 to 0.000) | 0.936 |
| Total cholesterol | -0.002 (-0.006 to 0.002) | -0.003 (-0.007 to 0.001) | 0.936 |
| LDL-C | -0.000 (-0.005 to 0.003) | -0.001 (-0.003 to 0.001) | 0.936 |
| HDL-C | -0.006 (-0.012 to 0.000) | -0.005 (-0.011 to 0.002) | 0.936 |
| Apolipoprotein A | -0.002 (-0.008 to 0.003) | -0.003 (-0.010 to 0.002) | 0.936 |
| Apolipoprotein B | 0.000 (-0.004 to 0.004) | 0.000 (-0.002 to 0.001) | 0.936 |
| Cystatin C | 0.001 (-0.001 to 0.004) | 0.001 (-0.004 to 0.007) | 0.936 |
| Direct bilirubin | 0.002 (-0.003 to 0.011) | -0.001 (-0.011 to 0.003) | 0.936 |
| GGT | 0.023 (-0.001 to 0.059) | 0.025 (-0.009 to 0.066) | 0.936 |
| Oestradiol | 0.008 (-0.058 to 0.141) | 0.001 (-0.019 to 0.045) | 0.936 |
| Rheumatoid factor | 0.009 (-0.036 to 0.065) | 0.006 (-0.037 to 0.096) | 0.936 |
| Total bilirubin | 0.003 (-0.001 to 0.012) | 0.004 (-0.001 to 0.010) | 0.936 |

Abbreviations: IGF-1, insulin-like growth factor-1; SHBG, sex hormone-binding globulin; CRP, C-reactive protein; HbA1c, hemoglobin A1c; LDL-C, low-density lipoprotein cholesterol; HDL-C, high-density lipoprotein cholesterol; GGT, gamma-glutamyl transferase.

Models were adjusted for age, ethnicity, body mass index, smoking status, alcohol intake, physical activity, healthy diet, maternal smoking around birth, history of diabetes and hypertension. FDR was corrected using the Benjamini-Hochberg method.

**Supplementary Table 6. Sex differences in natural direct effects of candidate mediators**

| Mediator | Males, β (95% CI) | Females, β (95% CI) | FDR for difference |
| --- | --- | --- | --- |
| Oestradiol | 0.249 (-0.100, 0.823) | -0.699 (-1.008, -0.321) | 0.029 |
| IGF-1 | -0.078 (-0.188, 0.066) | -0.222 (-0.347, -0.085) | 0.161 |
| Albumin | -0.091 (-0.223, 0.007) | -0.257 (-0.406, -0.093) | 0.161 |
| Vitamin D | -0.084 (-0.217, 0.064) | -0.276 (-0.430, -0.096) | 0.161 |
| Total protein | -0.092 (-0.205, 0.031) | -0.284 (-0.489, -0.091) | 0.161 |
| Testosterone | -0.074 (-0.217, 0.086) | -0.316 (-0.490, -0.139) | 0.161 |
| Lipoprotein A | -0.014 (-0.179, 0.148) | -0.196 (-0.362, -0.046) | 0.161 |
| SHBG | -0.070 (-0.189, 0.080) | -0.259 (-0.433, -0.134) | 0.161 |
| Urea | -0.094 (-0.192, 0.027) | -0.266 (-0.407, -0.126) | 0.161 |
| C‑reactive protein | -0.067 (-0.230, 0.148) | -0.258 (-0.410, -0.116) | 0.161 |
| HbA1c | -0.066 (-0.192, 0.104) | -0.285 (-0.427, -0.126) | 0.161 |
| Urate | -0.079 (-0.244, 0.058) | -0.282 (-0.412, -0.062) | 0.161 |
| Triglycerides | -0.069 (-0.184, 0.079) | -0.269 (-0.448, -0.110) | 0.161 |
| Total cholesterol | -0.081 (-0.256, 0.040) | -0.246 (-0.349, -0.122) | 0.161 |
| LDL-C | -0.086 (-0.233, 0.053) | -0.259 (-0.399, -0.088) | 0.161 |
| HDL-C | -0.093 (-0.299, 0.070) | -0.281 (-0.433, -0.144) | 0.161 |
| Apolipoprotein A | -0.099 (-0.255, 0.029) | -0.274 (-0.407, -0.106) | 0.161 |
| Cystatin C | -0.088 (-0.263, 0.045) | -0.267 (-0.434, -0.102) | 0.161 |
| Direct bilirubin | -0.022 (-0.128, 0.102) | -0.272 (-0.409, -0.087) | 0.161 |
| GGT | -0.047 (-0.167, 0.095) | -0.241 (-0.345, -0.111) | 0.161 |
| Total bilirubin | -0.064 (-0.208, 0.061) | -0.257 (-0.405, -0.092) | 0.161 |
| Calcium | -0.085 (-0.228, 0.119) | -0.258 (-0.378, -0.097) | 0.163 |
| Phosphate | -0.096 (-0.261, 0.091) | -0.280 (-0.468, -0.135) | 0.163 |
| Apolipoprotein B | -0.086 (-0.245, 0.102) | -0.244 (-0.397, -0.102) | 0.179 |
| Alkaline phosphatase | -0.079 (-0.228, 0.063) | -0.237 (-0.426, -0.102) | 0.178 |
| Creatinine | -0.095 (-0.270, 0.045) | -0.258 (-0.436, -0.107) | 0.178 |
| Glucose | -0.098 (-0.267, 0.150) | -0.283 (-0.457, -0.143) | 0.178 |
| Rheumatoid factor | -0.248 (-0.512, 0.042) | -0.100 (-0.414, 0.267) | 0.509 |

Abbreviations: IGF-1, insulin-like growth factor-1; SHBG, sex hormone-binding globulin; CRP, C-reactive protein; HbA1c, hemoglobin A1c; LDL-C, low-density lipoprotein cholesterol; HDL-C, high-density lipoprotein cholesterol; GGT, gamma-glutamyl transferase.

Models were adjusted for age, ethnicity, body mass index, smoking status, alcohol intake, physical activity, healthy diet, maternal smoking around birth, history of diabetes and hypertension. FDR was corrected using the Benjamini-Hochberg method.

**Supplementary Table 7. Association between breastfeeding in infancy and serum IGF-1 levels by sex**

| Males, β (95% CI) | Females, β (95% CI) | P for interaction |
| --- | --- | --- |
| -0.05 (-0.12, 0.01) | 0.10 (0.05, 0.16) | 0.039 |

Models were adjusted for age, ethnicity, body mass index, smoking status, alcohol intake, physical activity, healthy diet, maternal smoking around birth, history of diabetes and hypertension.

**Supplementary Table 8. Association between serum levels of IGF-1 and severe liver disease by sex**

|  | Quartile | HR (95% CI) | P value | P for interaction |
| --- | --- | --- | --- | --- |
| Males | Q1 | 1.00 (reference) |  | 0.056 |
|  | Q2 | 0.35 (0.31, 0.40) | <0.001 |  |
|  | Q3 | 0.28 (0.24, 0.32) | <0.001 |  |
|  | Q4 | 0.28 (0.24, 0.32) | <0.001 |  |
| Females | Q1 | 1.00 (reference) |  |  |
|  | Q2 | 0.46 (0.39, 0.54) | <0.001 |  |
|  | Q3 | 0.35 (0.28, 0.42) | <0.001 |  |
|  | Q4 | 0.34 (0.28, 0.42) | <0.001 |  |

Models were adjusted for age, ethnicity, body mass index, smoking status, alcohol intake, physical activity, healthy diet, maternal smoking around birth, history of diabetes and hypertension.

**Supplementary Table 9. Association of breastfeeding in infancy with MASLD, MASH, cirrhosis, HCC, and liver-related mortality in adulthood when excluding participants with missing covariates**

|  | **Males** | | **Females** | | **P for interaction** |
| --- | --- | --- | --- | --- | --- |
|  | Being breastfed in infancy | | Being breastfed in infancy | |  |
|  | No | Yes | No | Yes |  |
| MASLD by PDFF >5.5% |  |  |  |  |  |
| Model 1^1^ OR (95% CI) | 1 (Reference) | 0.95 (0.86-1.06) | 1 (Reference) | 0.91 (0.80-1.02) | 0.600 |
| Model 2^1,2^ OR (95% CI) | 1 (Reference) | 0.97 (0.87-1.08) | 1 (Reference) | 0.93 (0.83-1.04) | 0.583 |
| Model 3^1,2,3^ OR (95% CI) | 1 (Reference) | 0.98 (0.87-1.10) | 1 (Reference) | 0.94 (0.83-1.07) | 0.628 |
| MASH by PDFF >5.5% and cT1 >800ms |  |  |  |  |  |
| Model 1^1^ OR (95% CI) | 1 (Reference) | 0.98 (0.80-1.21) | 1 (Reference) | 0.75 (0.59-0.94) | 0.088 |
| Model 2^1,2^ OR (95% CI) | 1 (Reference) | 1.00 (0.81-1.24) | 1 (Reference) | 0.77 (0.60-0.97) | 0.087 |
| Model 3^1,2,3^ OR (95% CI) | 1 (Reference) | 1.07 (0.86-1.34) | 1 (Reference) | 0.77 (0.60-0.99) | 0.046 |
| Cirrhosis |  |  |  |  |  |
| Model 1^1^ HR (95% CI) | 1 (Reference) | 0.94 (0.83-1.06) | 1 (Reference) | 0.68 (0.59-0.79) | 0.009 |
| Model 2^1,2^ HR (95% CI) | 1 (Reference) | 1.04 (0.90-1.19) | 1 (Reference) | 0.74 (0.63-0.87) | 0.015 |
| Model 3^1,2,3^ HR (95% CI) | 1 (Reference) | 1.00 (0.86-1.17) | 1 (Reference) | 0.78 (0.64-0.94) | 0.082 |
| HCC |  |  |  |  |  |
| Model 1^1^ HR (95% CI) | 1 (Reference) | 1.16 (0.87-1.56) | 1 (Reference) | 0.54 (0.36-0.80) | 0.003 |
| Model 2^1,2^ HR (95% CI) | 1 (Reference) | 1.17 (0.85-1.61) | 1 (Reference) | 0.53 (0.33-0.84) | 0.004 |
| Model 3^1,2,3^ HR (95% CI) | 1 (Reference) | 1.17 (0.82-1.67) | 1 (Reference) | 0.54 (0.31-0.93) | 0.016 |
| Liver-related mortality |  |  |  |  |  |
| Model 1^1^ HR (95% CI) | 1 (Reference) | 1.02 (0.83-1.26) | 1 (Reference) | 0.58 (0.45-0.77) | 0.001 |
| Model 2^1,2^ HR (95% CI) | 1 (Reference) | 1.22 (0.97-1.54) | 1 (Reference) | 0.70 (0.51-0.97) | 0.003 |
| Model 3^1,2,3^ HR (95% CI) | 1 (Reference) | 1.22 (0.93-1.59) | 1 (Reference) | 0.86 (0.58-1.26) | 0.044 |

Abbreviations: MASLD, metabolic dysfunction-associated steatotic liver disease; MASH, metabolic dysfunction-associated steatohepatitis; HCC, hepatocellular carcinoma; HR, hazard ratio; CI, confidence interval.

^1^Adjusted for age and ethnicity.

^2^Adjusted for income, education, and Townsend deprivation index.

^3^Adjusted for body mass index, smoking status, alcohol intake, physical activity, healthy diet, maternal smoking around birth, history of diabetes and hypertension.

**Supplementary Table 10. Association of breastfeeding in infancy with incident cirrhosis, HCC, and liver-related mortality in adulthood using landmark 2-year analysis**

|  | **Males** | | **Females** | | **P for interaction** |
| --- | --- | --- | --- | --- | --- |
|  | Being breastfed in infancy | | Being breastfed in infancy | |  |
|  | No | Yes | No | Yes |  |
| Cirrhosis |  |  |  |  |  |
| Events No./total No. | 264/39268 | 768/120887 | 227/65366 | 394/153084 |  |
| Model 1^1^ HR (95% CI) | 1 (Reference) | 0.82 (0.71-0.95) | 1 (Reference) | 0.67 (0.56-0.79) | 0.066 |
| Model 2^1,2^ HR (95% CI) | 1 (Reference) | 0.88 (0.76-1.01) | 1 (Reference) | 0.71 (0.60-0.84) | 0.049 |
| Model 3^1,2,3^ HR (95% CI) | 1 (Reference) | 0.89 (0.77-1.03) | 1 (Reference) | 0.71 (0.60-0.84) | 0.089 |
| HCC |  |  |  |  |  |
| Events No./total No. | 44/39276 | 185/120929 | 32/65378 | 48/153108 |  |
| Model 1^1^ HR (95% CI) | 1 (Reference) | 0.98 (0.70-1.37) | 1 (Reference) | 0.49 (0.31-0.78) | 0.019 |
| Model 2^1,2^ HR (95% CI) | 1 (Reference) | 1.02 (0.73-1.42) | 1 (Reference) | 0.51 (0.32-0.81) | 0.019 |
| Model 3^1,2,3^ HR (95% CI) | 1 (Reference) | 1.00 (0.71-1.39) | 1 (Reference) | 0.52 (0.33-0.83) | 0.028 |
| Liver-related mortality |  |  |  |  |  |
| Events No./total No. | 95/39085 | 317/120199 | 73/65118 | 101/152666 |  |
| Model 1^1^ HR (95% CI) | 1 (Reference) | 0.94 (0.74-1.18) | 1 (Reference) | 0.54 (0.40-0.74) | 0.004 |
| Model 2^1,2^ HR (95% CI) | 1 (Reference) | 1.02 (0.80-1.29) | 1 (Reference) | 0.60 (0.44-0.82) | 0.003 |
| Model 3^1,2,3^ HR (95% CI) | 1 (Reference) | 1.04 (0.82-1.32) | 1 (Reference) | 0.62 (0.46-0.85) | 0.008 |

Abbreviations: HCC, hepatocellular carcinoma; HR, hazard ratio; CI, confidence interval.

^1^Adjusted for age and ethnicity.

^2^Adjusted for income, education, and Townsend deprivation index.

^3^Adjusted for body mass index, smoking status, alcohol intake, physical activity, healthy diet, maternal smoking around birth, history of diabetes and hypertension.

**Supplementary Table 11. Association of breastfeeding in infancy with accidental death**

|  | **Males** | | **Females** | | **P for interaction** |
| --- | --- | --- | --- | --- | --- |
|  | Being breastfed in infancy | | Being breastfed in infancy | |  |
|  | No | Yes | No | Yes |  |
| Events No./total No. | 76/39237 | 247/120809 | 72/65325 | 171/153001 |  |
| Model 1^1^ HR (95% CI) | 1 (Reference) | 0.84 (0.65-1.09) | 1 (Reference) | 0.81 (0.61-1.07) | 0.956 |
| Model 2^1,2^ HR (95% CI) | 1 (Reference) | 0.90 (0.69-1.17) | 1 (Reference) | 0.82 (0.62-1.09) | 0.988 |
| Model 3^1,2,3^ HR (95% CI) | 1 (Reference) | 0.89 (0.69-1.16) | 1 (Reference) | 0.84 (0.63-1.11) | 0.945 |

Abbreviations: HR, hazard ratio; CI, confidence interval.

^1^Adjusted for age and ethnicity.

^2^Adjusted for income, education, and Townsend deprivation index.

^3^Adjusted for body mass index, smoking status, alcohol intake, physical activity, healthy diet, maternal smoking around birth, history of diabetes and hypertension.
